# Case Report: Labor induced coccydynia associated with Modic I changes successfully treated with platelet-rich plasma

**DOI:** 10.3389/fimmu.2023.1239741

**Published:** 2023-10-30

**Authors:** Miloš Vuković, Igor Nosek, Jelena Vuković, Đorđe Ilić, Duško Kozić, Jasmina Boban

**Affiliations:** ^1^ Department of Radiology, Faculty of Medicine Novi Sad, University of Novi Sad, Novi Sad, Serbia; ^2^ Department for Radiology Diagnostics, Oncology Institute of Vojvodina, Sremska Kamenica, Serbia; ^3^ Department of Gynaecology and Obstetrics, Faculty of Medicine Novi Sad, University of Novi Sad, Novi Sad, Serbia; ^4^ Obstetrics and Gynaecology Clinic, Clinical Center of Vojvodina, Novi Sad, Serbia

**Keywords:** edema, inflammation, pain, vertebral endplates, coccyx

## Abstract

Imaging can aid in determining potential causes of coccygeal pain and therefore guide clinicians to carry out individualized treatment. We represent a case of postpartum coccydynia treated by platelet-rich plasma (PRP) which was assessed and followed by MRI. A primipara with uncomplicated labor developed coccygeal pain after delivery that significantly limited her postpartum recovery. On the first MR scan, recorded 6 months after delivery, there were edematous changes of the vertebral endplates of Co1-4 level (Modic type I) with the presence of pronounced precoccygeal venous drainage. Degenerative changes with signs of edema in the area of the pubic symphysis were recorded. The sacroiliac joints had regular morphological features. Since the patient was breastfeeding, PRP therapy was applied with a total of three injections in the area of the coccyx subcutaneously, once every 3 months. The subjective feeling of pain reduction after each injection was about 30%, with the complete withdrawal of pain after one year and still pain-free at the two-year follow-up. One year after the initial MR imaging, a follow-up MR examination was performed, where almost complete resolution of edematous changes in the previously present zones was observed, with residual minor edema of the vertebral endplates at the Co2-3 level. Edema of the pubic bones in the area of the pubic symphysis also subsided. A case of labor-induced coccydynia that was represented as Modic type I changes without neither fracture or luxation was successfully treated with PRP with complete resolution of symptoms.

## 1 Introduction

Being the insertion site for multiple muscles, ligaments, and tendons, the coccyx provides support to a person in a seated position, as well as positional support to the anus. It also forms the posterior border of the pelvic outlet, making it an essential factor for vaginal birth.

Coccydynia may be a consequence of either external or internal trauma. While external trauma usually occurs due to a backward fall, internal injury develops during childbirth, especially during a difficult or instrumented delivery. Nontraumatic coccydynia can result from several causes, including degenerative joint or disc disease, hypermobility or hypomobility of the sacrococcygeal joint, infectious etiology, and variants of coccygeal morphology. Factors associated with an increased risk of developing coccydynia include obesity and female gender (1).

Imaging can aid in determining potential causes of coccygeal pain and therefore guide clinicians to carry out individualized treatment. Radiological findings associated with coccydynia include edema of the intercoccygeal joint and the adjacent vertebral endplates, fracture and subluxation, hypermobility of the intercoccygeal joint with edema in and adjacent to the joint, the presence of liquid collection in the joint, T2W STIR hyperintense structure at the tip of the coccyx probably representing inflammation in the soft tissues, as well as the presence of similar structure dorsal to the coccyx in a patient with spicula most likely compatible with a bursa (2). To the best of our knowledge, there is only one published study investigating radiological findings in cases of postpartum coccydynia in which the diagnostic modality of choice was dynamic radiography (3). However, in almost 40% of cases, dynamic radiography failed to prove any significant changes that would explain a cause of pain.

We represent a case of postpartum coccydynia which was assessed and followed by MRI. In this paper, we also discuss the new therapeutical approach in patients with coccydynia and their effectiveness on follow-up.

## 2 Case description

The patient was primipara at 37 weeks of gestation and admitted to a maternity hospital after the spontaneous onset of labor, with a completely effaced cervix and dilatation of 4cm at the admission. After 5 hours of an active phase of labor and 30 minutes of an expulsion stage, the patient gave birth to a baby boy with a birth weight of 3430g. The delivery was complication-free, without the usage of either forceps or a suction cup. The episiotomy was performed. However, the child had a cephalhematoma. The pain in the area of the coccyx was minimal during the pregnancy itself, with significant intensification after childbirth. After delivery, the patient complained of pain in this area that raised suspicion of a hematoma as a consequence of a birth trauma, which was then ruled out. She also could not sit, which significantly limited her postpartum recovery. During pregnancy, there was also pain in the area of the pubic symphysis with pain intensification when lifting weights.

**Figure 1:**
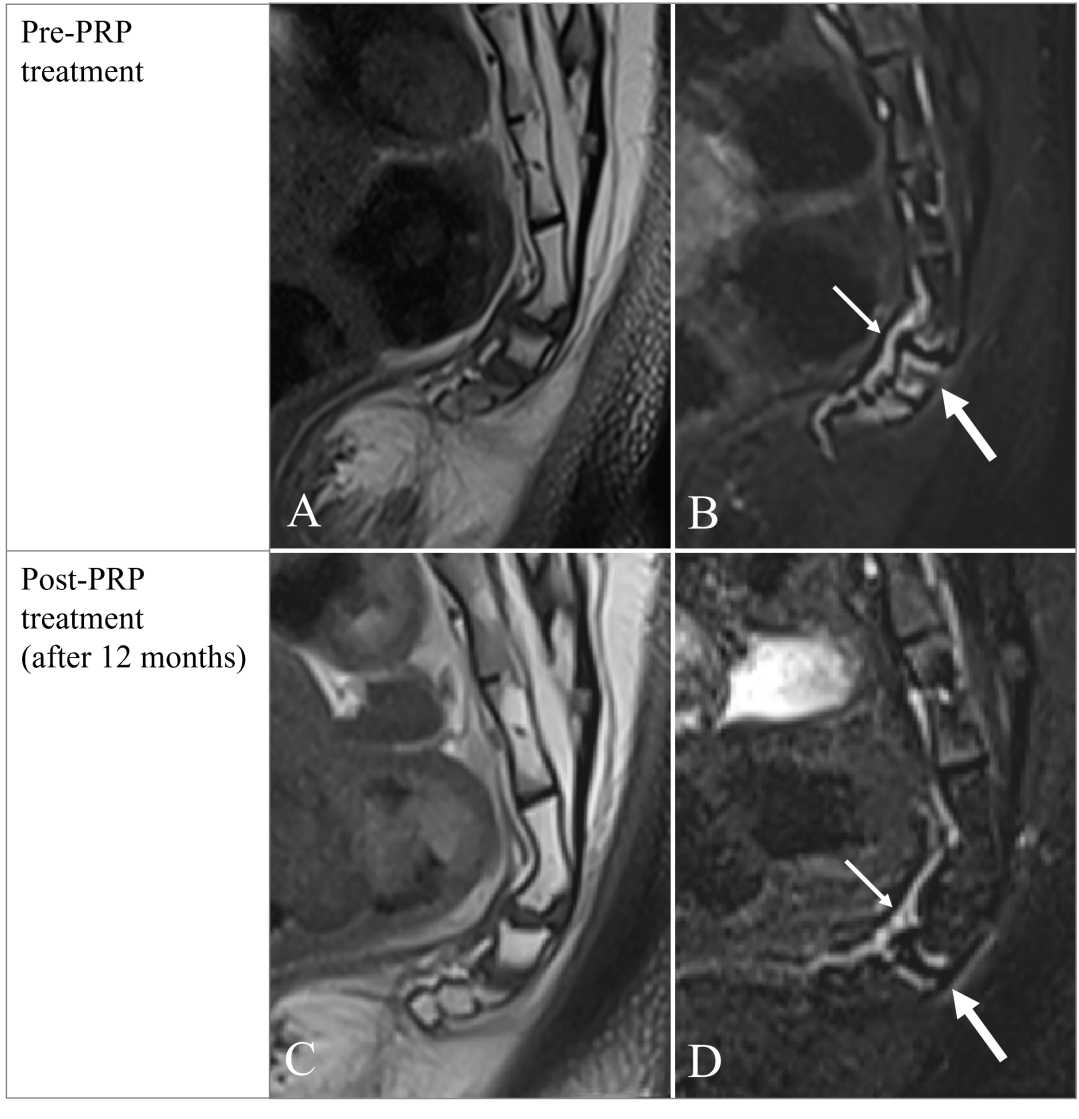
Initial MR examination (**(A)**-T1W; **(B)**-TIRM) shows edema of vertebral endplates of the coccyx (**(B)**-thick arrow) with pronounced large drainage vein (**(B)**-thin arrow) on the ventral aspect of the coccyx; follow-up one year after initial examination (**(C)**-T1W; **(D)**-TIRM) shows reduction in volume and diameter of drainage vein on the ventral aspect of the coccyx (**(D)**-thin arrow) and only little residual edema of Co2-3 vertebral endplates (**(D)**-thick arrow).

**Figure 2:**
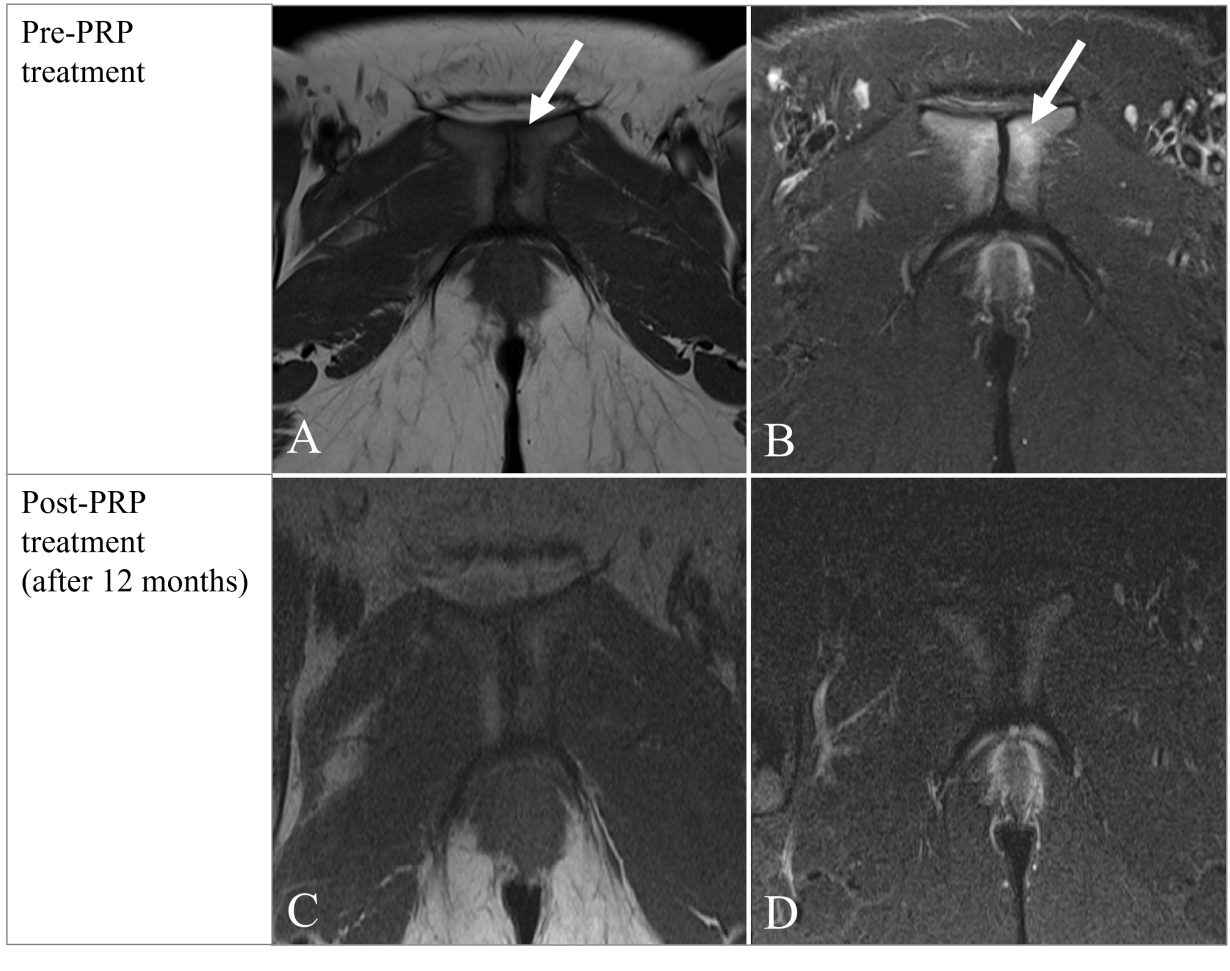
Initial MR examination (**(A)**-T1W; B-TIRM) shows irregular articular surfaces of pubic symphysis with small erosions and edema (**(A, B)** arrow) indicating degenerative changes; follow-up one year after initial examination (**(C)**-T1W; **(D)**-TIRM) shows complete resolution of edema of articular surfaces of the pubic symphysis.

On the first MRI scan, recorded 6 months after delivery, the pubococcygeal diameter recorded on the pre-therapy MR examination was 8cm. On that examination, there were edematous changes of the vertebral endplates of Co1-4 level with the presence of pronounced precoccygeal venous drainage (Modic type I) (**Figures 1A, B**). Degenerative changes with signs of edema in the area of the pubic symphysis were recorded (**Figures 2A, B**). The sacroiliac joints had regular morphological features.

The patient tried a conservative treatment that included an exercise program of reverse Kegels, core strengthening, and reduction of time spent in a sitting position. That did not result in long-term relief of pain. After consultation with the physiatrist and considering that the patient was breastfeeding, the decision was made to try PRP (platelet-rich plasma) instead of classical local corticosteroid therapy.

Thirty milliliters of whole blood was processed with an Emcyte Pure-PRP system (EmCyte Corporation, FL, USA) to produce 3 ml of leukocyte-poor PRP. After sterile preparation, PRP was delivered into the posterior sacrococcygeal tendon. The patient received a total of 3 injections of PRP in the area of the coccyx subcutaneously, once every 3 months. The subjective feeling of pain reduction after each injection was about 30%, after the first 30%, the second by about 60%, and 9 months after the third injection pain reduction subjectively amounted to about 90%. After one year, the patient was pain-free and maintained without pain at the two-year follow-up.

One year after the initial MR imaging, a follow-up MR examination was performed, where almost complete resolution of edematous changes in the previously present zones was observed, with residual minor edema of the vertebral endplates at the Co2-3 level (**Figures 1C, D**). Edema of the pubic bones in the area of the pubic symphysis also subsided (**Figures 2C, D**).

## 3 Discussion

This paper describes the rare form of the pathological substrate of coccygodynia that occurred after childbirth, as well as the specificity of the therapeutic approach in this unique case.

Case reports of coccygodynia published so far in the literature describe the use of corticosteroid therapy and its effectiveness. Considering that the use of corticosteroids is not desirable in pregnant and breastfeeding women, we have followed the effectiveness of PRP therapy in an unusual form of coccygodynia after childbirth.

Coccydynia is a common syndrome characterized by pain localized to the tailbone that radiates into the lower sacrum and perineum (4). So far reported cases of postpartum tailbone pain were associated with either fracture, luxation, or hypermobility of the coccyx (3). In our case, neither fracture nor luxation was found. Instead, there were edematous changes of the vertebral endplates of Co1-4 level with the presence of pronounced precoccygeal venous drainage (Modic type I) (**Figures 1A, B**). The presence of drainage veins may indicate slight inflammation at the tip of the coccyx. In one published study (2), drainage veins were found almost exclusively in patients with a rigid coccyx. As in the mentioned study, our patient also had pronounced drainage veins, which suggests that the coccyx was probably also rigid and represented an obstacle to the increase of the pelvic outlet, which led to trauma to the coccyx and also the occurrence of cephalhematoma. Although the restricted pubococcygeal diameter of 8cm could be a predisposing factor to childbirth trauma, it is well known that adequate molding of the pelvis i.e. mobility of pelvic joints provides non-complicated labor even in these circumstances. Therefore, the rigid coccyx might have led to birth trauma and coccydynia in our patient. Degenerative changes with signs of edema in the area of the pubic symphysis were also recorded (**Figures 2A, B**), indicating new stress changes resulting from increased body weight. The sacroiliac joints had regular morphological features.

Regarding the treatment, traditional corticosteroid injections have become an established therapeutic option among those who treat coccydynia, but occasionally, at the site of injection, skin, and soft tissue atrophy may occur with long-acting corticosteroids such as triamcinolone (5). The benefit of a corticosteroid injection is somewhat variable, some report barely 3 months of pain-free, and others did not report any pain 9 months after treatment (6). If pain persists, alternative options are usually ganglion impar block and radiofrequency. There is no strong evidence to support coccygectomy (4).

One study compared the association of the severity of inflammatory endplate changes identified on MRI and clinical response to intradiscal corticosteroid injection in nonspecific chronic low back pain (6). It found that the reduction in pain score after one month was significantly higher in patients exhibiting Modic I signal changes than in those with Modic II signal changes (7). This was evidenced by an accelerated switch from Modic I to Modic 0 signal changes, as seen on lumbar MRI at 1-month follow-up (6). This indicates that early treatment in patients with coccydynia results in better therapeutical response and attention must be dedicated to approaching every patient individually.

As previously mentioned, pathologic laxity of the sacrococcygeal ligament can also be a cause of coccydynia, as well as ligamentous laxity of the first intercoccygeal joint. In cases of painful hypermobility and ligamentous laxity throughout the body, the use of PRP injections has been reported as a successful method of treatment (8).

The biopsy studies of cases with Modic type I of vertebral endplates showed the molecular background of these changes, that is replacement of vertebral endplate bone marrow with richly vascularized fibrous tissue (9), increased levels of interleukin-6 (IL-6) (10), and a higher number of tumor necrosis factor (TNF) immunoreactive cells (11). The way that PRP treatment supports reparative processes and wound healing is through the distribution of a wide range of growth factors and proteins in the affected area. These proteins are platelet-derived growth factors (PDGF), transforming growth factor–β (TGF-β), vascular endothelial growth factor (VEGF), epidermal growth factor (EGF), and adhesive proteins – fibrin, fibronectin, and vitronectin. Each of these proteins plays a specific role in this complex process of tissue reparation (12). PRP treatment is therefore only an accelerator of the natural healing process in these patients, which makes it a safe and successful treatment option.

PRP for the treatment of coccydynia was recently reported by Montero-Cruz and Aydin (2018), who demonstrated decreased pain in three subjects after a fluoroscopic-guided PRP injection into the deep and superficial sacrococcygeal ligaments (13). In another study in which PRP was used, at the 6-week follow-up, the patient reported 70% pain relief and had total resolution of pain with usual activity and sitting on cushioned surfaces. At 6 months post-injection, her coccydynia had completely resolved. At the 12-month follow-up, she remained pain-free similar to our case (8).

## 4 Conclusions

This article presents a cause of new-onset coccygodynia after childbirth in the form of MRI-documented edematous changes in the vertebral endplates, which indicates its association with trauma. The report emphasizes the success of PRP treatment for the postpartum recovery of breastfeeding women for whom traditional corticosteroid therapy is not recommended. The effectiveness of PRP was confirmed objectively by a control MRI scan. The only drawback of this type of treatment is its higher price, but it is a therapeutic option that should be pursued in the future because it safely promotes the natural recovery process.

## Data availability statement

The original contributions presented in the study are included in the article/supplementary material. Further inquiries can be directed to the corresponding author.

## Ethics statement

Ethical approval was not required for the study involving humans in accordance with the local legislation and institutional requirements. Written informed consent to participate in this study was not required from the participants or the participants’ legal guardians/next of kin in accordance with the national legislation and the institutional requirements. Written informed consent was obtained from the individual(s) for the publication of any potentially identifiable images or data included in this article.

## Author contributions

MV and IN contributed to the concept and design, data collection, and writing of the manuscript. JV and ĐI contributed to the gynecological aspect of the paper, by acquiring needed measurments and data. DK and JB contributed to the final draft. All authors contributed to the article and approved the submitted version.
